# Integrating Public Health Into Undergraduate Medicine in North America: A Systematic Review

**DOI:** 10.7759/cureus.36284

**Published:** 2023-03-17

**Authors:** Muhammad Uzair Khalid, Omar Mahboob, Shawn Khan, Farah Naaz Manji, Jasmine Pawa

**Affiliations:** 1 Temerty Faculty of Medicine, University of Toronto, Toronto, CAN; 2 Dalla Lana School of Public Health, University of Toronto, Toronto, CAN; 3 Florida State University College of Medicine, Florida State University, Tallahassee, USA; 4 Department of Anesthesiology and Pain Medicine, University of Toronto, Toronto, CAN

**Keywords:** global health, curriculum design, medical education, population health, public health

## Abstract

The coronavirus disease 2019 (COVID-19) pandemic has served as a stark reminder of the importance of foundational public health training for all physicians. However, the most effective way to incorporate these concepts into undergraduate medical education remains unclear. Here, we characterize the literature regarding the effectiveness of public health integration into undergraduate medical education in North America.

We systematically searched MEDLINE, Embase, Cochrane Central, and Education Resources Information Center (ERIC) in accordance with preferred reporting items for systematic review and meta-analysis (PRISMA) guidelines for North American peer-reviewed literature, published from 01/01/2000 to 30/08/2021, that described outcomes of integrating public health training within an undergraduate medical curriculum. Results were qualitatively synthesized into key themes.

A total of 38 studies, involving interventions across 43 medical schools, were included. Studies reported on a combination of public (n=13), global (n=9), population (n=9), community (n=6), and epidemiological (n=1) health interventions, and either implemented one-off workshops, electives, or international experiences (n=19); a longitudinal theme or long-term enrichment pathway (n=14); or a case-based learning curriculum (n=8). The majority (81.5%, 31/38) of integrations were self-described as successful and, of studies reporting on feasibility, most (94.1%, 16/17) were indicated as feasible. The definition of what constituted such success, however, was unclear. Innovative examples included the use of simulation workshops and mobile-optimized media content. Key challenges were noted, however, in securing adequate funding and buy-in from administrative leadership. Robust community partnerships and iterative cycles of implementation of the intervention were critical factors to success.

In summary, foundational public health components can be effectively integrated into medical school curricula and would benefit from adequate resourcing, innovation, community partnerships, and continuous improvement.

## Introduction and background

Health systems have both health care and public health components. Whereas the former of these encompasses all medical and surgical interventions provided to patients on an individual basis, the latter of these has been contentiously defined in the literature. Building on past definitions, the Canadian Public Health Association, for example, defines public health as "the organized effort of society to keep people healthy and prevent injury, illness and premature death" [1,2].

Yet, historically in Canada, health care has accounted for approximately 95% of all governmental funding, with public health receiving closer to 5% [3,4]. Moreover, World Health Organization (WHO) assessments of public health teaching in medical education around the world show that public health concepts are de-emphasized and not tested as frequently as other medical concepts in standardized testing [5]. This is despite public health and population health being a foundational competency for all healthcare providers, including both physicians and, as increasingly emphasized by the Medical Council of Canada, medical students [6]. Moreover, the coronavirus disease 2019 (COVID-19) pandemic has further highlighted the need for robust public health systems and appropriate training for physicians and other healthcare providers to be able to interact with public health systems and public health service delivery [7,8].

Certainly, there is growing recognition of the importance of integrating public health concepts within the core curriculum for all physicians in training [9-11]. Medical schools have increasingly expanded public health lectures to include videoconferences, interactive workshops, and integrated case studies [12]. More recently, medical schools have revised their curricula to allow medical students to participate in electives and/or field placements to learn public health concepts in the context of the current pandemic [7,8]. Yet, to date, no systematic assessment exists to characterize the effectiveness of these teaching strategies for undergraduate medical education (UME) students. 

As such, this systematic review seeks to characterize the literature regarding public health curricula in UME. Specifically, we aim to characterize the types of public health integrations into the medical curricula that are both successful and feasible, as well as the thematic key factors for success that have driven these projects.

This article was previously presented as a poster and abstract presentation at the 2022 Ontario Student Medical Education Conference on April 2, 2022.

## Review

Methods

Pre-defined search terms were used to search four scientific databases from inception to August 30, 2021. Included databases were Ovid MEDLINE®, Ovid Embase®, Cochrane Central, and Education Resources Information Center (ERIC). The complete search strategy is outlined in Table 1.

**Table 1:** Literature search strategy. Searches conducted in Ovid MEDLINE®, Ovid EMBASE®, Cochrane Library, and ERIC databases are shown, along with their associated yields. ERIC: Education Resources Information Center.

Database	Search strategy	Yield
Ovid MEDLINE®	Public health/ or Global Health/ or Population Health/ or Community Medicine/ or Epidemiology/ or Preventative Medicine/ or Public Health Practice/ Education, Medical, Undergraduate/ Course content/ or curriculum/ or curriculum development/ 1 and 2 and 3 ((public health or community health or population health) adj3 (undergraduate medic* or UME or medic* student* or medic* train* or preclerkship* or pre clerkship*)).tw,kw. 4 or 5	656
Ovid Embase®	Exp Public health/ or Global Health/ or Population Health/ or Public Health Practice/ or Community Medicine/ or “Social determinants of health”/ Medical education/ or clinical education/ Course content/ or curriculum development/ or curriculum/ 1 and 2 and 3 ((public health or community health or population health or epidemiology) adj3 (undergraduate medic* or UME or medic* student* or medic* train* or clerkship* or preclerkship* or pre clerkship*)).tw,kw.	1639
Cochrane Central	Public health or Global Health or Population Health or Community Medicine or Epidemiology or Preventative Medicine or Public Health Practice Course content or curriculum development or curriculum 1 and 2	26
ERIC	Public health or Global Health or Population Health or Community Medicine or Epidemiology or Preventative Medicine or Public Health Practice Course content or curriculum development or curriculum 1 and 2	32

Searches aimed to include all original, peer-reviewed studies in English that described or evaluated an intervention in a medical school curriculum that enabled the integration of public health education into that curriculum. Public health education was defined to also include epidemiology, preventive medicine, population health, global health, or community health. Later, search criteria were further narrowed for relevancy to only include papers published from 01 January 2000 onward as well as those originating from North American medical schools. Posters and abstract presentations were excluded.

Included literature was screened via a protocol concordant to the preferred reporting items for systematic review and meta-analysis (PRISMA) guidelines. This included a title and abstract screening, followed by a full-text review and subsequent data extraction step. All stages were independently completed by two authors, with a third reviewer resolving discrepancies with mutual consensus. Data extraction was done via Microsoft Excel® (Microsoft Corporation, New York, USA) and included information on the study design and names of stakeholder medical schools, the self-described domain of public or population health, the objectives of the curricular intervention, a description of the type of intervention, numerical outcomes (if any), self-declared success and feasibility, and any overarching conclusions from the study. 

Results

**Figure 1:**
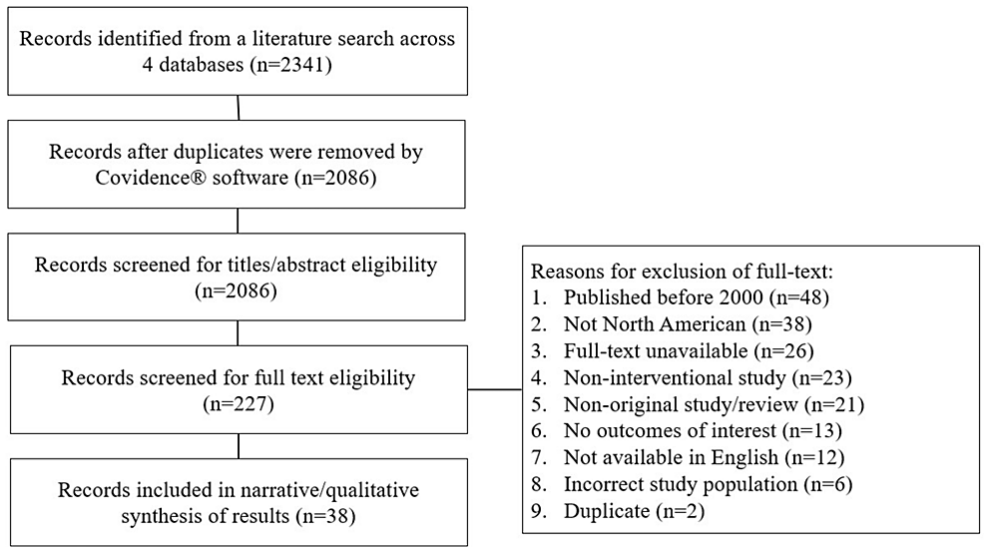
PRISMA flowchart. The number of manuscripts screened, excluded, and selected at each step are outlined. PRISMA: preferred reporting items for systematic review and meta-analysis.

The search strategy yielded a total of 2341 manuscripts across four databases (Table 1). After the removal of 255 duplicates, title and abstract screening was performed on 2086 studies for relevance to the search criteria. With 227 manuscripts subsequently undergoing full-text screening, 38 studies were finally included for data extraction and result synthesis, as shown in Figure 1 [7,9-11,13-46]. An abbreviated consensus table of the extracted data is provided in Table 2.

**Table 2:** Abbreviated findings from papers. The location and nature of the public health integrations, any numeric or qualitative outcomes, and overarching conclusions from each study are summarized. Each intervention is also summarized in a few words preceding the full description, with "course" generally describing a discrete educational opportunity, and a "curriculum" being a longitudinal component of the medical school training. Success and feasibility were both defined by the original authors themselves and reported here as a quote. Y: yes; N: no; NR: not reported.

Citation	University	Description of curriculum integration	Outcomes	Successful and/or feasible? (Y/N/NR)
Abu-Odeh et al. (2017) [13]	The City University of New York-School of Medicine	Course: The second-year course was mandatory for all students and aimed to develop physicians who could apply population health perspectives to community-based health programs. The course contained 13 content modules; interactive lectures; real-life applications via short videos; weekly workshops; and social, epidemiological, ecological, educational, and policy assessment assignments.	Overall, students rated the course favorably. Suggestions for improvements were linking content to a grade, linking lectures to workshops, and to clearly delineate the assignments in the course.	Successful: Y–“Coupling population health concepts with practical application supports students’ engagement and understanding.” Feasible: NR.
Altillo et al. (2021) [7]	The University of Texas at Austin Dell Medical School	Two to four-week elective: The elective, designed to create a service-learning opportunity to train in pandemic response, had two main components: an online platform with asynchronous modules, readings, discussion boards, and group presentations; and a pandemic response field placement. The elective was offered to upper-year students in three formats: (1) enrichment elective (online); (2) two-week elective (online + placements); or (3) four-week elective (online + placements + capstone project).	The most favorably reviewed aspects of the course were the field placements, the opportunity to learn from peers, self-directed research, and the resources suggested by the course faculty. Students reported increased confidence around limited personal protective equipment, limited ventilators, and fear in the setting of COVID-19 infection.	Successful: Y–"The elective was well-received by students, achieved stated objectives, and garnered public attention." Feasible: Y–"This educational innovation could serve as a model for medical schools globally."
Bales et al. (2018) [14]	Wright State University Boonshoft School of Medicine	One-month elective international experience: Students were sent to a community partner in Swaziland, where they offered medical outreach to the local population 3×/week in rural, mountainous areas. They worked in one of four areas: triage/human immunodeficiency virus testing, operating rooms, distributing donated shoes, or shadowing a general practitioner. Medical students also designed research projects. Objectives included increasing cultural awareness, understanding different health care systems, and functioning in low-resource settings.	A pre-departure curriculum improved student’s self-reported knowledge of tropical medicine, cultural awareness, culture shock, and resource availability. Students presented posters at over ten meetings, and four articles were accepted or under consideration for publication. No post-placement quantitative assessments of knowledge were conducted to support the conclusions.	Successful: Y–"The educational experience of medical students is enhanced through the interaction of delivering patient care and completing clinical research studies." Feasible: NR.
Bertelsen et al. (2015) [15]	New York University School of Medicine	Four-week selective:A selective was offered annually as five activities: clinical assignments at a local hospital; cultural competency and clinical skills simulations; case discussions in tropical medicine; literature review and journal clubs; and lectures. The aim was to develop cross-cultural communication skills and learn about social determinants of health and global health disparities.	Thirty-three students participated over three years, with the majority of students in third-year. Qualitative evaluation by students was consistently positive, including in cultural competence, ethics, social determinants, and health disparities. Case discussions and experiential learning workshops received the highest ratings.	Successful: Y–"Experiential, student-centered teaching employed in this course proved successful." Feasible:NR.
Brill et al. (2011) [16]	University of Wisconsin School of Medicine and Public Health	Mapping longitudinal objectives: Public health competencies were mapped to each clerkship’s current educational objectives to determine existing linkages. Objectives were to negotiate short- (didactic and experimental) and long-term goals to incorporate public health competencies.	The clerkship directors' response was largely positive, credited to (1) offering choices of curricular focus; (2) dean-level support for the initiative; (3) strong interpersonal relationships among the directors and public health team; and (4) team efforts to support and resource the process.	Successful: Y–"Outcomes (…) include successful initiation of experiential learning activities." Feasible: Y–"The process might easily be replicated at other sites."
Buckner et al. (2010) [17]	Morehouse School of Medicine	Course: All students in the medical program conducted a community health needs assessment and then developed, evaluated, and presented a community health promotion intervention. The course aimed to deliver information around community health analysis, health-related behavior, and community health promotion. Instructional methods include hands-on community activities in a small group setting, assignments, and lectures.	Over 500 students conducted 56 community interventions in the first 11 years of the course. Interventions included educational programs, health fairs, and policy initiatives.	Successful: Y–"Provided students with an opportunity to obtain collaborations with diverse communities to address community health." Feasible: Y–"The (course) is a model that other medical schools (…) can use."
Campos-Outcalt (2011) [9]	University of Arizona College of Medicine	Longitudinal theme:The theme aimed to provide public health content in the form of lectures and case-based instructions. Each basic science block included a public health lecture on the most common conditions in the block as well as associated risks, disparities, and prevention. Public health electives were also available in fourth-year.	No outcomes were reported.	Successful and feasible: NR.
Carney et al. (2011) [10]	Four: (1) Case Western Reserve University, (2) Harvard Medical School, (3) the University of Colorado School of Medicine, and (4) University of Vermont College of Medicine	Workshops: (1) At Case Western, a six-hour workshop was held as part of a five-week population health block, which included a pandemic influenza tabletop exercise. (2) At Harvard, a workshop was held as part of a first-year course. Groups of students were assigned a role, such as a local public health department or the mayor’s office, and were supported by a coach to act out a simulation. (3) At Colorado, a three-hour exercise was held as part of a required fourth-year course. The workshop was preceded by a lecture on emergency preparedness and followed by a debrief. (4) At Vermont, a one-hour introductory lecture was followed by a simulated emergency broadcast, with students in small groups acting out the simulation. All four institutions aimed to train students in emergency pandemic response.	Initiating planning well in advance of the exercise was essential to develop a case that was realistic, illustrated public health issues, highlighted the varied contributions of physicians as members of multidisciplinary teams, and involved community- and public-health-based participants and stakeholders. Most students at all institutions rated the simulation exercise positively.	Successful and feasible: Y–"The authors concur (...) that conducting preparedness exercises is one practical, engaging, and effective way to incorporate public health principles to medical school curricula."
Chamberlain et al. (2008) [11]	Stanford University School of Medicine	Curriculum/case-based learning: New lectures in a "Practice of Medicine" course covered social and economic determinants of health, health disparities, physician advocacy, and environmental health. Population health concepts and approaches were also integrated into case studies and discussions within the broader curriculum. First-year students were required to engage in a community-based population health project.	Barriers included the student perception that the experience constituted required volunteer work, and having insufficient time to devote to the projects. Most projects focused on disease prevention and health promotion, with a quarter focused on healthcare access issues. Project quality was not assessed/reported.	Successful: Y–“Experience to date shows that classroom-based sessions and experiential learning in the area of population health can be successfully integrated into a medical school curriculum." Feasible: N–"Although logistically challenging (..)."
Chamberlain et al. (2020) [18]	University of Illinois Chicago College of Medicine	Four-year global medicine program: The program was offered to select medical students as an enrichment, and included didactic instruction and workshops in global medicine, as well as exposure to supplementary content (e.g., cultural competency, economic perspectives of global aid, and the ethics of volunteerism) in alternative learning formats like film reviews, book club discussions, and simulation-based cases. Students also took part in two research projects, including a longitudinal capstone project.	About 74% of projects involved original research, and 71% involved international travel. About 26% of projects led to a conference abstract/presentation, while 14% led to a publication. The majority of projects focused on emergency medicine, obstetrics/gynecology, and primary care.	Successful: Y–"A structured global health capstone is one method for (...) preparing students for careers as global health practitioners and leaders." Feasible: Y–"Overall, we found that a four-year longitudinal capstone is feasible."
Dannenberg et al. (2002) [19]	Multiple	Electives: Electives lasting 4+ weeks were arranged at local health departments, and focused on infectious diseases, and outbreak investigations. Some electives included more didactic components or encouraged the student to publish a manuscript.	Cooperation between health departments and medical schools was necessary for medical student rotations. Essential elements included identifying a faculty in the medical school interested in public health and an epidemiologist or other professional at the health department interested in mentoring students.	Successful: Y–"Electives at health departments are critical to recruiting in the field of public health."Feasible: Y–"These electives can help guide additional medical schools and health departments as they initiate such rotations."
Velarde et al. (2007) [20]	University of New Mexico’s School of Medicine	Public health certificate: Faculty, in collaboration with community stakeholders, created a mandatory certificate equivalent to 15 graduate-level units. The courses aimed to cover principles of public health, epidemiology, biostatistics, evidence-based medicine, health systems, health policy, a community research project, and a public health elective.	Strategies for implementing an institution-wide public health certificate include: the use of a "logic model," building political support within the institution and the community, identifying stakeholders; establishing goals, identifying and assessing resources, and conducting evaluation.	Successful: NR. Feasible:Y–"Introduction of a public health certificate (PHC) for all students is feasible with planning tools such as the logic model."
Duffy et al. (2014) [21]	University of Oklahoma College of Medicine	One-week prematriculation immersion experience: The mandatory curriculum, designed to address disparities in health access and outcomes, was divided into appreciative inquiry teams, world cafe groups, large group activities, prototype development teams, small group activities, professional meaning groups, anchoring lectures, and poverty simulation and food stamps lunches.	Qualitative data show that students had powerful learning experiences during the experience. Community interviews, the poverty simulation, exposure to underserved patients in the clinic, and the relationships forged with faculty and other students were ranked as useful.	Successful: Y–"Evaluations show that the experience changes attitudes and bonds groups to enjoy interdisciplinary work while focusing on community health problems." Feasible:NR.
Evans et al. (2016) [22]	Stanford University School of Medicine	Curriculum: The mandatory quantitative medicine curriculum blended online self-paced learning with in-class collaborative learning, with the objective to understand research methods as it relates to disease, patient care, and public health. Its innovative, asynchronous online component provides opportunities for active and self-monitored learning. Students complete three online modules every two weeks, followed by a small-group case-based session.	Student responses revolved around accessibility, time neutrality, pace, additional learning aids, quizzes, and learning style preferences. There were significant improvements in student satisfaction, and most students reported using online videos as their primary learning resource.	Successful and feasible: Y–"Evaluation suggests that our blended quantitative medicine (QM) curriculum is successful and that its features could serve as a model for future blended courses."
Finkelstein et al. (2008) [23]	Harvard Medical School	Course: An intensive, mandatory, four-week block course was taught in the first year, with the objective of improving knowledge in epidemiology, biostatistics, and population-level health promotion, using large-group settings, conferences, and small-group tutorials.	The mean rating for the course overall was substantially better than the previous clinical epidemiology course, and the mean number of items answered correctly increased.	Successful: Y–"The first iteration of the course was well received, and assessments of students suggested mastery of basic skills."Feasible: NR.
Francis et al. (2012) [24]	Weill Cornell Medical College	Curriculum: The curriculum offered a comprehensive four-year elective pathway with 100+ hours of training, including three courses, two international experiences, a preceptorship with a clinician working with underserved populations, and regular lectures and seminars by visiting global health leaders. Topics include the global burden of disease, inequalities, human rights, research and evidence-based outcomes, and health systems delivery.	Overall, feedback was very positive, although some students desired an increased level of rigor to the courses and for structured mentorship throughout the program. Securing funding was noted as a consistent challenge.	Successful: Y–"This is a student initiative that, with faculty mentorship and administrative support, has yielded a formal, robust global health program." Feasible: Y–"This student-driven model (can) guide other medical schools."
Geppert et al. (2011) [25]	University of New Mexico School of Medicine	Public health certificate: The certificate was mandatory for all medical students, and included courses in "Health Equity: Principles of Public Health," "Epidemiology and Biostatistics Course," "Evidence-Based Practice Course," "Health Systems and Health Policy," "Community-Based Service Learning," and "Ethics and Public Health."	Overall, the faculty received good comments from students, including in the increased understanding of public health concepts, social determinants of health, the relationship between public health and medicine, and the identification of faculty mentors in community health.	Successful: Y–"The UNMSOM Public Health Certificate Program is an innovative approach to restoring medicine and public health to their historic Hippocratic unity." Feasibility: NR.
Godfrey et al. (2019) [26]	Columbia Vagelos College of Physicians and Surgeons	Commute curriculum:The mandatory five-week curriculum covered health systems, social determinants, race, substance use, violence, and alternative care models. Modules incorporated 30 minutes of mobile-optimized media content on a particular public health theme that students could conveniently interact with during their free time or their commute. Topics also included optional resources (e.g., documentaries and interviews) for interested students.	After completing the curriculum, a greater proportion of students acknowledged the impact of public health on their clinical practice, patient’s outcomes, and choice of a residency program or employment site.	Successful: Y–"Learners participating in this five-week online public health curriculum demonstrated a significant increase in public health knowledge." Feasibility: NR.
Gruner et al. (2015) [27]	Three: (1) University of Ottawa, (2) Memorial University of Newfoundland, and (3) University of Saskatchewan	Randomized control trial and focus groups: Students voluntarily completed an online knowledge quiz and a validated global health self-assessment questionnaire. They were then randomly assigned to either (a) the beta-version of the Refugees and Global Health eLearning Program, or (b) two global health education articles in PDF format on global health, ethics, and best practice guidelines. Results deemed by researchers as significant were used to inform focus groups, with the goal to introduce basic concepts of global health to medical students.	Three themes emerged regarding eLearning modules: (1) facilitators, (2) barriers, and (3) curriculum delivery. Participants highlighted their preference for eLearning to introduce new global health knowledge. They mentioned personal interest in global health, online convenience, and integration into the curriculum as incentives to complete the eLearning. Overall, there was an increase in conceptual knowledge in both groups.	Successful: Y–"Both the e-learning and the peer-reviewed PDF articles improved global health conceptual knowledge" Feasibility: NR.
Hoover et al. (2012) [28]	University of California, Berkeley-San Francisco Joint Medical Program	Problem-based learning cases: A public health case was added to the first-year curriculum, with the goal to motivate students to explore public health concepts and contemplate the physician’s role in promoting population health. Students self-generated learning issues and assigned topics for individual study, and then returned to class later to present material they had prepared individually as a written report and an oral presentation.	Of the 29 student-generated learning objectives, 20 contained information relevant to at least one of the 12 public health competencies. All 12 recommended ‘‘Public Health Competencies for Medical Students.’’	Successful: Y–"Problem-based learning (PBL) is a promising tool to enhance medical students’ engagement with public health." Feasibility:NR.
Izadnegahdar et al. (2008) [29]	All 17 in Canada	Questionnaire: Using a structured questionnaire, information was collected from deans’ offices, institutional representatives, faculty, students, and medical school websites to understand the quality and quantity of global health educational activities at Canadian medical schools.	There was no uniform approach regarding the year of delivery, topics covered, or amount of information in global health teaching across Canadian medical schools. No undergraduate medicine program provided a mandatory, stand-alone credit course in global health. Electives ranged from two-year programs with didactic components and electives in a developing country to just two to three hours of lectures on global health-related issues.	Successful: NR, Feasible: NR.
Johnson et al. (2011) [30]	University of Toronto	Curriculum: The Determinants of Community Health course incorporated public health education across all four years of the mandatory medical curriculum in a spiral fashion. In first-year, definitions of health, the determinants of health, the role of community agencies, and health promotion were taught. In second-year, students completed a research project. In third-year, evidence-based medicine, quality improvement, patient safety, outbreak management, and legal aspects of medicine were taught. In fourth-year had a capstone week after a year of clinical ward experiences.	The evaluation of lectures and tutorials in community health was equivalent to those of other courses. In terms of scores on the licensing exam, the University of Toronto was ranked either first or second place nationally, in comparison to lower rankings in previous years.	Successful:Y–"The course has been successfully run since 1999." Feasible: Y–"For the same amount of curricular time, an integrated spiral curriculum for teaching public health appears to be more effective than traditional approaches."
Kasper et al. (2016) [31]	Harvard Medical School	Courses: A four-course mandatory sequence was implemented: Introduction to Social Medicine and Global Health; Health Policy; Clinical Epidemiology and Population Health; and Medical Ethics and Professionalism. This included weekly lectures, readings, and case-based tutorials.	About 64% of students reported a deeper understanding of social medicine concepts and their relevance for clinical practice in the US and abroad, 81% felt better equipped to conduct a social history, and 80% stated that social medicine was very important to their overall education.	Successful: Y–"Such a course can equip medical students with the knowledge and tools that they will need to address complex health problems." Feasible: Y–"Can be used at other medical schools."
Magill et al. (2001) [32]	University of Utah School of Medicine	Course: The primary care preceptorship was six weeks long and required for fourth-year medical students. The students worked 60% of the time in a primary care practice in a medically underserved community. For the other 40%, students designed a community health project in consultation with the physician, the local health department, and hospital administrators.	Some students objected to the required rather than elective nature of the rotation. Others disliked the need to make temporary moves to other towns. Students completed projects on clinical problems, community health assessments, patient education, and epidemiology.	Successful: Y–"The primary care preceptorship (PCP) demonstrates the successful introduction of community health into a clinical curriculum." Feasible: NR.
Maurana et al. (2000) [33]	Medical College of Wisconsin	Partnership: The center followed four stages of development in their partnership with a rural community with the goal of community health improvement: (1) establish/build relationships, (2) develop common goals, (3) develop/implement programs, and (4) maintain/expand progress. Students made mandatory presentations to high school students about their experiences as medical students and gave health-related demonstrations.	Medical students had positive experiences and were exposed to rural medicine with its numerous challenges and benefits.	Successful:Y–"The success of the center’s partnership (…) was determined (…) by the partners’ ability to (…) work through the four stages of partnership development." Feasible: NR.
McCurdy (2003) [34]	University of California at Davis Medical School	Case-based learning cases: A required four-week course in second-year was created called “Principles of Epidemiology and Preventive Medicine,” with both lectures and case-based learning.	Students were concerned that they were missing important points as they had not been provided a list of correct answers.	Successful: NR. Feasible: NR.
McIntosh et al. (2008) [35]	University of Rochester School of Medicine and Dentistry	Clerkship: Students could take either a four-week intensive clerkship in public health (mandatory) or a longitudinal experience across four years. Longitudinal students participated in community health activities and didactic online modules. Intensive students got didactic lectures from local agencies, read through supplemental online modules, and developed community health projects.	Focus-group comments revealed that students viewed the clerkship positively and that they found it to be valuable to their respective career paths. Most students said their project improved the health of the social group or community with which they interacted.	Successful: Y–"This successfully meets the recommended changes in medical education." Feasible: Y–"This clerkship has demonstrated its feasibility."
Meurer et al. (2011) [36]	Medical College of Wisconsin	Optional pathway: Students spent 10+ hours/month on pathway activities: four hours on core material delivered through readings, didactics, case discussions, and site visits; and 6+ hours of experiential non-core activities, guided by an individualized learning plan and a faculty advisor. Non-core activities include community-engaged research, service-learning activities, and submission of a synthesis paper. The goal was to cover public health, social determinants, cultural humility, poverty, the local healthcare system, and safety nets.	Students enjoyed working with peers across classes and favored interactive, community-based sessions over classroom didactics. They were frustrated over a perceived increase in workload and a lack of clarity in expectations. Projects included public health assessments, analysis of population health data, and community-based health promotion.	Successful: Y–"The Curriculum Evaluation Committee (CEC) review indicated that the (program) was successful in its fırst year." Feasible: NR.
Nackers et al. (2020) [37]	University of Wisconsin School of Medicine and Public Health	Course:Curriculum was divided into three phases. In phase 2, a 12-week course included didactic content from required blocks and the creation of new content on public health in the context of historical pandemics. Phase 3 students were rescheduled into online electives and participated in a public health preparedness course. Objectives included public health ethics, health equity, population dynamics, and outbreak control and surveillance.	For phase 2, students found the delivery method at least equivalent to the face-to-face format they had experienced before. For phase 3, students appreciated the opportunity to engage in a timely and relevant topic.	Successful: Unclear–"it is premature to determine the success of these efforts (but) we are poised for positive outcomes." Feasible: NR.
Pearson (2003) [38]	University of Rochester School of Medicine and Dentistry	Case-based learning cases:The following required cases were presented: community health assessment, infant mortality, colon cancer screening, tuberculosis in a homeless men’s shelter, adolescent sexually transmitted diseases, and adolescent suicide prevention.	Overall, the performance composite score decreased from at post-test. The satisfaction of the students shows scores somewhat lower than that found in SUNY–Upstate students but in a similar range (fair to good).	Successful: N– "Evaluation (…) revealed no change in population-oriented skills." Feasible: Y–"Cases are readily implemented.”
Pottie et al. (2007) [39]	University of Ottawa	Course: The voluntary program was composed of an Internet-based training module and a self-assessment quiz focused on global and refugee health, a workshop to increase competence in cultural matters, an experience working with at least one refugee family at a shelter for newly arriving refugees, a family physician mentorship, and a debriefing workshop at the end.	Refugees said that the outreach allowed their questions to be answered, helped them understand Canada’s healthcare, and made their first visits to doctors easier. Physicians reported that families who had gone through the interview had a better understanding of the system and were more interested in preventive services. Students reported an improvement in their knowledge and skills for brokering cross-cultural health care.	Successful:Y–"Working at a community refugee shelter provided a powerful learning experience for students." Feasible: NR.
Premkumar et al. (2010) [40]	University of Saskatchewan	Voluntary modules: Instructors used four Public Health Agency of Canada modules in their course: Basic Epidemiological Concepts, Measurement of Health Status, Descriptive Epidemiological Methods, and Outbreak Investigation and Management. Changes were made to improve relevance to medical students, and the four modules were converted into one “supercourse," delivered as a combination of virtual and face-to-face sessions.	Students felt that the information was useful and relevant, and liked the flexibility of the online component. Yet, they were dissatisfied with the videos/audios as they addressed public health professionals and disliked the amount of information/assessments. Instructors felt that making modifications was time-consuming. All stakeholders felt the need for more time, proper project design, and role specification.	Successful: N–"Using online content originally developed for a different target audience (…) did not prove to be an effective learning experience." Feasible: Y–"It is feasible to repurpose the online modules in undergraduate medical education."
Prunuske et al. (2017) [41]	University of Wisconsin School of Medicine and Public Health	Activity: Students conducted a guided community health assessment as part of their required six-week community medicine rotation in fourth-year, which included a description of community members’ perceptions of health problems, identification of a single priority health issue, an evidence-based intervention, a written report, and a workshop presentation.	Student-rated skill in conducting a community health intervention increased as did student-rated skill in finding evidence that supports public health programs and policies. There was no difference between the pre-test and post-test in the likelihood of participating in a community health assessment (CHA) organized by others, or in initiating a CHA as a practicing physician.	Successful: Y–"(This) improved students self-rated skill in conducting a community health assessment." Feasible: Y–"The addition of a community health intervention to a clinical rotation is feasible."
Schapiro et al. (2011) [42]	University of Wisconsin School of Medicine and Public Health	Cases: All students were divided into groups, with each group exploring one aspect of a case, such as basic science, clinical care, population health, and social/ethical issues by meeting with patients, advocates, community leaders, scientists, and physicians. They would then reunite and share their findings to make connections across each of their domains, with the objective to provide students with more knowledge around integrating public health into clinical medicine.	Comments from fırst- and second-year students highlighted the cases’ value and the importance of public health in their education.	Successful:Y–"Initial findings from this pilot indicate that integrative cases are an effective vehicle to incorporate public health concepts within the traditional basic and clinical science model of undergraduate medical education." Feasible: NR.
Sheline et al. (2014) [43]	Duke University School of Medicine	Curriculum: The four-year primary care leadership track required select students to contribute to existing community health initiatives, perform community-engaged research, and participate in leadership training. In addition, students regularly interacted with faculty to explore population health issues, review patient cases, and adjust individual learning opportunities as needed.	Students reported that they most appreciated the close relationships they cultivated with their longitudinal faculty and the experiences that occurred when they followed patients over time.	Successful: NR. Feasible: NR.
Stebbins et al. (2011) [44]	University of Pittsburgh School of Medicine	Area of concentration: The four-year optional stream included didactic sessions, leadership training, faculty mentorship, a scholarly project, a minimum one-month experiential component at a health department, and self-evaluation, with the aim of improving the visibility of public health concepts in the medical school. Students also participated in the program steering committee.	Third- and fourth-year medical students who completed the practicum highly rated the experience. Pre-/post-assessments of knowledge change showed an increase over 14 content areas. Students reported being “more interested” in careers in public health as a result of the experience.	Successful: Y–"The (program) has shown early success." Feasible: Y–"The public health (program) is a simple model for incorporating many key aspects of public health into medical education and can be duplicated by any university."
Sutphen (2003) [45]	State University of New York–Upstate Medical University.	Case-based learning cases: The mandatory preventive medicine instruction incorporated eight cases from the case-based series in population-oriented prevention, which covered community health assessments, health disparities, outbreak investigation, preventive screening, sexually transmitted diseases, helmet safety, and suicide prevention. There were also three lectures on epidemiology and biostatistics and three critical appraisal sessions.	Students’ mean performance on the skills and competency instrument increased. Just over half of the students also rated the cases as excellent or good.	Successful: Y–"The students demonstrated a significant increase in skills and competencies in preventive medicine." Feasible:NR.
Turner et al. (2008) [46]	College of Human Medicine at Michigan State University	Curriculum: A review of the curriculum was carried out to incorporate objectives related to poverty, barriers to health care, and chronic health conditions. Changes were made to the mandatory first- and second-year clinical skills program. New case scenarios were added with the help of a folklorist. Students were assigned to an individual with a chronic health condition, who they visited eight times over 14 months. Some third-year clerkships and two fourth-year rotations were changed as well.	Most anecdotes indicated that students had learned something meaningful from their encounters with low-socioeconomic status (SES) patients. Program directors rated graduates as above or substantially above average compared with other residents with respect to cultural competence, ability to work with low-income/Medicaid patients, and regarding the use of resources.	Successful: Y–"Our evaluation tells us that we did indeed successfully revise the curriculum to help increase medical graduates’ awareness of and ability to mitigate inequities in health care." Feasible: NR.

The majority of the 38 included studies were from the USA (n=33), with the remainder originating from Canada (n=5). This represented projects at 43 North American undergraduate medical school curricula, including all 17 medical schools in Canada. Of these, there were four papers from the University of Wisconsin School of Medicine and Public Health; three papers each from Harvard Medical School, the University of Ottawa, and the University of Saskatchewan; and two papers each from the Stanford University School of Medicine, the University of New Mexico’s School of Medicine, the Medical College of Wisconsin, the University of Rochester School of Medicine and Dentistry, the University of Toronto, and the Memorial University of Newfoundland. The remaining faculties of medicine were only represented once.

Studies reported on a combination of public (n=13) [9,10,16,19,20,25,26,28,32,37,40,42,44], global (n=9) [7,14,15,18,24,27,29,31,39], population (n=9) [11,13,23,34,38,41,43,45,46], community (n=6) [17,21,30,33,35,36], and epidemiological (n=1) [22] health interventions. Definitions of each of these were found to be highly overlapping; when reported, objectives of projects, although targeting different “types of public health,” were similar and often included social determinants of health (e.g., poverty, language skills, provider bias, and racism), population-level approaches to medicine, public policy, global burdens of disease, and pandemic preparedness. This reflected the multi-component coverage of public health teaching and practice. 

**Figure 2:**
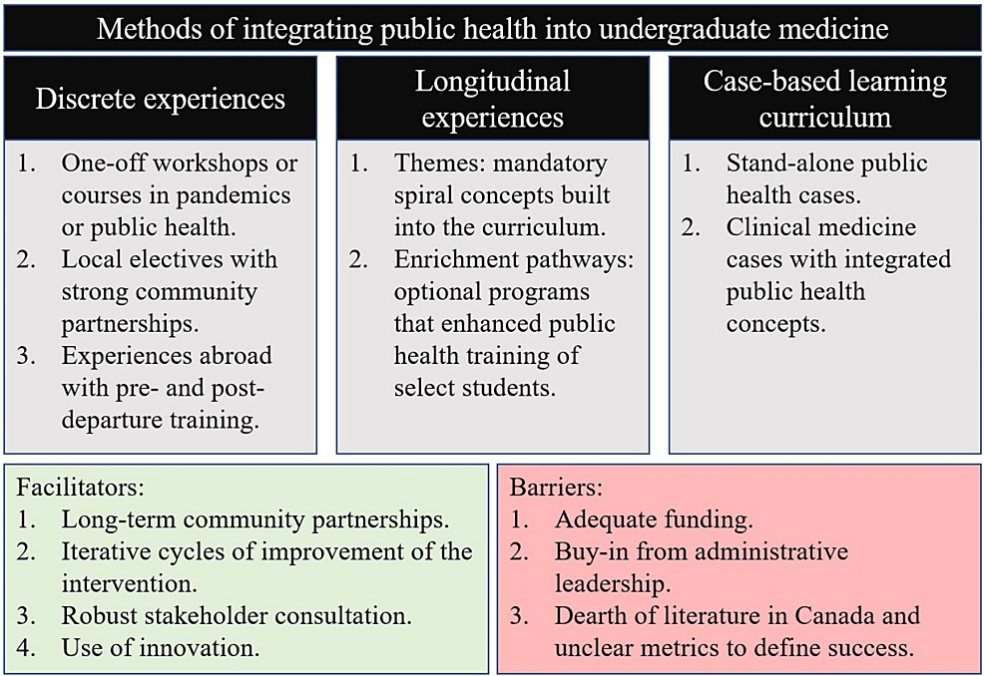
Public health can be integrated with undergraduate medicine as either a discrete experience, a longitudinal experience, or a case-based curriculum. Facilitators and barriers to these interventions, as outlined in the literature, are shown.

Of the interventions implemented by medical schools, we found three broad categories emerging, with some institutions having components across categories (Figure 2).

The first of these interventions included one-off workshops, simulation exercises, or clerkship electives (50.0%, n=19/38) [7,10,13-15,17,19,21-23,26,27,29,31,32,37,39-41]. These interventions were often characterized as being stand-alone, time-limited, and isolated in the curriculum, with the rest of the four years being largely unchanged. In general, students appreciated the opportunity these provided for real, albeit limited, immersion via community interviews and public health projects. 

The second category of intervention was the implementation of a longitudinal theme or long-term enrichment pathway throughout all four years of the medical school curriculum (36.8%, n=14/38) [9,11,16,18,20,24,25,30,33,35,36,43,43,44,46]. Themes were typically a mandatory part of the curriculum, whereas enrichment pathways allowed for select groups of students to enroll in programs that enhanced their public health training. These had two broad advantages. First, it allowed for the consistent reinforcement of medical student knowledge in a spiral-like curriculum, improving licensing exam outcomes [30,46]. Second, certificates allowed for students to enrich their medical school training with a certificate or faculty-endorsed qualification that they could use in their curriculum vitae. When designing these pathways, students at Stanford University, for example, appreciated the “accessibility, time neutrality, pace, additional learning aids, quizzes, and accommodation of learning style preferences” [22].

The last broad category of intervention, although similar to the first category in its length of time but distinct in its type of delivery, was a case-based learning curriculum (CBL, 21.1%, n=8/38) [9,11,28,34,36,38,42,45]. Whereas these served as an introduction to public health principles, this type of intervention was both the least studied and had the least successful outcomes. Two (25.0%) studies reported no outcomes about their case studies [9,11], and three (37.5%) studies reported generally negative outcomes [34,36,38], including the students’ desire to get the “right answers” at the end so that they are not missing anything [34], as well as a belief that content could not have been mastered without the core lectures [36]. Despite these negative comments, five out of eight studies describing a CBL still concluded their interventions as "successful," suggesting inconsistencies in what they deemed as success.

Indeed, of the 33 studies reporting on success, a staggering 93.9% (n=31/33) of public health integrations were self-described as successful by the authors. Two interventions were explicitly described as being unsuccessful. Premkumar et al. at the University of Saskatchewan described an unsuccessful attempt to repurpose Public Health Agency of Canada (PHAC) modules for medical students, reflecting a need for more purposefully designed content [40]; Pearson at the University of Rochester School of Medicine and Dentistry described a failed attempt to include CBL cases designed to meet public health objectives, citing a reduction in knowledge scores post-intervention, with no clear explanation behind such a decline [38]. 

On the other hand, among studies that reported on feasibility and/or cost-effectiveness, 94.1% (n=16/17) were indicated as feasible, and readily implementable at other medical schools. Chamberlain et al., however, described their overhaul of their curriculum to include new lectures along with case studies and community health projects as logistically challenging; despite this, they termed their intervention as providing great value to students [11].

Our thematic analysis identified four critical factors in success. Firstly, there needed to be robust long-term community partnerships, as exemplified by Maurana, which delineated four stages of partnership development: establishment and building of relationships, development of common goals, development and implementation of programs, and maintenance and expansion of progress [33]. Such partnerships needed to be bi-directional in their advantages, benefiting both students, as well as the community. Secondly, there needed to be iterative cycles of implementation and refinement of the intervention, including needs assessment, planning, implementation, and evaluation steps [11]. A third factor for success was strong planning and stakeholder/student consultation stages. Many projects enlisted student feedback to guide further success, with one study giving students the opportunity to be on the steering committee of the public health area of concentration [44] and another study describing an intervention that grew entirely out of a student-driven program of ad-hoc lectures [24].

A fourth factor underlying successful integrations was the use of innovation. One example included the use of immersive simulations in the form of multi-day workshops at Harvard Medical School, Case Western Reserve University, the University of Colorado School of Medicine, the University of Vermont College of Medicine, and the University of Wisconsin School of Medicine and Public Health [10,42]. At the former four schools, students acted out the roles of different stakeholders in public health, including politicians and the press, as they navigated a public health issue and were given updates as time progressed. These did, however, require a fair bit of planning. Another instance of innovation included the use of mobile-optimized media content as a 30-minute “commute curriculum” that students could access while traveling [26]. This intervention had great success, with 54% of students improving their score of public health concepts by a mean difference of 23.8% (p<0.001). A third example of innovation was the systematic mapping of current curricula onto public health objectives, allowing faculty leadership to collaborate and maximize buy-in while identifying areas where public health principles were already in play [16,46]. At the University of Wisconsin School of Medicine and Public Health, this exercise successfully initiated experiential learning activities in all but one required clerkship rotation, as well as didactics in all clerkships [16]. At the College of Human Medicine (CHM) at Michigan State University, this allowed for changes in 25% of the pre-clinical courses, all third-year clerkship rotations except psychiatry, and fourth-year rotations in surgery and internal medicine [46].

Key challenges were also noted, especially in securing adequate funding, as well as buy-in from administrative leadership [17,24,26,42,46]. Funding was usually described as a joint venture, coming from the medical college administration, short-term grant funding, local departments of health, and stakeholder partners like global health offices. As these become scarcer, many authors express concerns about the long-term sustainability of the project: “Will the faculty (...) sustain the revisions in the curriculum now that the project is officially over and external funding has ended” [46]? 

Finally, for Canadian medical schools, there was a dearth of literature on current and past integrations in public health. Indeed, the latest study that we found was Gruner from 2015 [27]. In this study, students enrolled in a randomized control trial to self-study global health via either PDF or eLearning modules; both groups had the same post-test outcomes, thereby discrediting the superiority of eLearning over a simple PDF. Additionally, a survey from 2008 showed that there was no uniform approach to the curriculum content in global health, with some schools devoting a mere two hours across the four years to the topic [29].

Discussion

This systematic review describes the current state of public health curricula integrations within the undergraduate medical education system in North America, as reflected in the academic literature. While there are a range of approaches employed and successes described, information on outcomes is limited. Challenges were described in getting buy-in for public health as a part of medical education. This is all also occurring in the context of broader changes to the medical education system.

The public health curricula presented throughout the undergraduate medical education system varied with respect to content. While the majority of programs reported the delivery of public health education, other programs framed their curricula in terms of population, community, and epidemiological health. While we recognize that public health is not synonymous with global health, a few papers describing global health curricula were also included as they came up in the review.

The delivery of the curricula was heterogeneous between institutions. Most institutions reported on the implementation of stand-alone programs that were administered at a singular point during the undergraduate medical education. These programs were time-limited. Other institutions employed longitudinal delivery, which often extended across the span of the undergraduate medical education. Compared to the stand-alone curricula, this mechanism of delivery assisted with reinforcement throughout the educational training of the students. Case-based delivery of content was the least reported in the literature and had the worst outcomes, despite its growing popularity within the undergraduate medical system. In general, two types of cases were reported: those designed solely as public health cases versus those where public health concepts were integrated with clinical medicine, with both types showing mixed results. Further research on the outcomes of case-based learning in public health education is needed to clarify the value of integration within curricula.

The importance of evaluating the success of the curricula was underscored in the literature. Most of the studies self-reported on whether the intervention was considered successful. However, there were no standardized metrics used to evaluate success. Most studies used a combination of quantitative and qualitative data to gauge the perspectives of students and the learning that occurred throughout the intervention. A clear definition of interventions and outcomes (i.e., “what is success” in this area) would be helpful when comparing different interventions for implementation. Certainly, the authors believe that such success could be heterogeneously assessed, such as via the accomplishment of "entrustable professional activities" within a competency-based curriculum, overall licensing exam scores, residency match rates, or the qualitative yet cumulative assessment of preceptors. 

Feasibility and cost-effectiveness of interventions did not feature in much of the literature; however, most studies that reported on this measure found their intervention to be feasible and cost-effective. Barriers to implementation were typically logistical and included faculty time and compensation constraints. Buy-in from administrative and healthcare leadership for public health education specifically was also described as a challenge. More information on if and how misunderstandings about public health practice within the health care sections of our system contribute to these challenges may be helpful. 

Establishing authentic longitudinal partnerships within the community was considered to be crucial in the success of many programs. Many programs also voiced the importance of structured program design, including assessment, planning, implementation, and evaluation. Stakeholder consultation was promoted as a mechanism for obtaining evaluative data and included feedback from both community partners, faculty, and medical students.

The combination of innovation and collaboration was featured in some interventions. Partnerships between different institutions to provide immersive experiences for medical students enabled the interventions to capture the most current and applicable public health scenarios. Increased recognition of the value of partnerships in the delivery of public health curricula requires the further development of roles and responsibilities and the allocation of funding to ensure equitable access. The importance of collaboration within an institution was also identified to be crucial in maximizing buy-in. Leveraging existing non-public health curricula to integrate public health objectives is an opportunity to seamlessly deliver content within an established course.

All of this discussion is happening in a broader context of recommended reforms to healthcare provider education, including for medical students, as outlined in a recent Lancet Commission [47]. At its core, health is about individuals (and their communities) and their needs; health professional education must respond to these needs. The COVID-19 pandemic has further reinforced the long-standing need for well-developed public health systems, which needs to be reflected in medical education. We can also expect more challenges to population health, such as from climate change, in the future. Some have argued that health professional education has not kept up with health and health complexities with an educational model that is inflexible and out-of-date, with clear opportunities for reform [47]. Improving the integration of public health skills into medical education is one of these important opportunities. 

Limitations

To the authors’ knowledge, this is the first systematic review that looks at the success and feasibility of public health education integration into the medical education curriculum. However, there are several limitations to the work that was performed here. Firstly, the quality of the included literature was not evaluated, given the heterogeneity in study design, reporting of outcomes, and manuscript style. Secondly, both success and feasibility were declared by the authors, instead of being based on some objective measures, with unclear criteria of what constituted either. This makes the assessment of metrics challenging in a realistic context, especially when most of the studies described in this review delineate early findings and new projects, with sparse long-term sustainability data. Finally, given the broad, evolving definitions of public health, and our restriction to literature published in North America in the English language, there are likely studies missing from this review. 

## Conclusions

Results of this systematic literature review indicate that public health curricula in the North American undergraduate medical system are heterogeneous in their content and delivery. Clear and iterative planning; long-term partnerships with communities, students, faculty, and administrative leadership; a commitment to innovation; and use of immersion and innovation can improve student uptake, feedback, and perceived success of the integration. Additional work is needed in evaluating program success and feasibility, incorporating calls for broader reforms in medical and healthcare provider education.
